# Comparison of *In Vitro* and *In Situ* Methods for Studying Lipolysis

**DOI:** 10.1155/2013/205385

**Published:** 2013-08-19

**Authors:** Ahmad Ghorbani, Mahmood Abedinzade

**Affiliations:** ^1^Pharmacological Research Center of Medicinal Plants, School of Medicine, Mashhad University of Medical Sciences, Mashhad 9177948564, Iran; ^2^Paramedical School of Langeroud, Guilan University of Medical Sciences, Langeroud 4193844937, Iran

## Abstract

Lipolysis is a highly regulated process and is controlled by nervous system, hormones, and paracrine/autocrine factors. Dysregulation of lipolysis is associated with some pathophysiological conditions including diabetes, metabolic syndrome, and obesity. Nowadays, special attention isthereforepaid to study lipolysis using different experimental models. This review summarizes the current experimental methods for studying lipolysis. Culture of preadipocyte cell lines, use of differentiated stroma-vascular cells, primary culture of adipocyte, organ culture of adipose tissue, and microdialysis technique are the most widely used techniques to study lipolysis. The advantages and limitations of using these methods are discussed.

## 1. Introduction

Adipose tissue is one of the largest body compartments with various physiological roles including lipid handling and hormone secretion. It is responsible for the storage of fat as triglyceride (via lipogenesis) during times of energy excess and for the mobilization of triglyceride (via lipolysis) during periods of calories deficit [1]. Hormone-sensitive lipase, a rate-limiting enzyme, and monoglyceride lipase catalyse hydrolysis of triglyceride to release fatty acids and glycerol. Unlike fatty acids, glycerol is not reutilized by adipocytes because these cells contain very little glycerol kinase [2]. Therefore, measurement of glycerol gives a good estimate for level of lipolysis [3]. Lipolysis is a highly regulated process and is disciplined by nervous system, hormones (e.g., insulin and catecholamines), and paracrine/autocrine factors (e.g., adenosine, prostaglandins, nitric oxide, and TNF-*α*) [1, 3–5]. Physical activity, nutrition, gender, age and genetic are also important determinant factors for lipolysis [2, 4]. 

Dysregulation of lipolysis is associated with a number of pathophysiological conditions such as obesity, diabetes, metabolic syndrome, familial combined hyperlipidaemia, and polycystic ovarian syndrome [4]. Nowadays, special attention is therefore paid to study lipolysis and other aspects of adipose tissue metabolism. While isotopic tracer techniques and arteriovenous difference method allow *in vivo* estimation of lipolysis, culture of preadipocyte cell lines, primary culture of adipocyte, organ culture of adipose tissue, and microdialysis are of the most used techniques for studying lipolysis *in vitro* and *in situ*. This review summarizes the *in vitro* and *in situ* techniques for studying lipolysis in animal and humans. Moreover, the advantages and drawbacks of using each method are discussed (Table 1).

## 2. Experimental Methods

### 2.1. Culture of Preadipocyte Cell Lines

There are several murine and human preadipocyte cell lines for studying adipocyte metabolism, for example, 3T3-L1, 3T3-F422A, LS14, LiSa-2, and HPB-AML-I [6–9]. Among them, 3T3-L1 cells are best characterized and widely used in lipogenesis and lipolysis research for over 30 years. Originally, the cells were isolated from Swiss 3T3 mouse embryos by Green and Kehinde [6]. Preadipocyte cell lines have fibroblast-like morphology. However, when they enter, a confluent stage undergoes a differentiation to an adipocyte-like phenotype by addition of a standard differentiation cocktail containing insulin, dexamethasone, and a nonselective phosphodiesetrase-3 inhibitor [10]. These cells are sensitive to lipolytic (e.g., *β*-adrenergic receptor agonists) and antilipolytic (e.g., insulin) agents [10, 11]. Upon reaching confluence, 3T3-L1 cells start to express markers characteristic of differentiation from day 3 and usually complete the differentiation process by day 8 [10–12]. Therefore, it took more than one week until the cells be ready for lipolysis study. Yet, the cells often remain multilocular (multiple lipid droplets) and may not fully differentiate to unilocular adipocytes. Further, differentiation capacity of 3T3-L1 cells decreases with increasing number of passages [13]. In addition to other limitations of cell culture technique, results from *in vitro* studies may not fully reflect *in vivo* condition due to physiologic responses active in the whole organism. On the other hand, one advantage of this method is that preadipocytes are adherent cell lines and therefore are suitable for studying molecular mechanisms and transcription factors involved in lipolysis process. Also, culture of these cell lines provides an abundant supply of homogeneous cells which make it an appropriate model for screening compounds for their possible lipolytic or antilipolytic effects. Furthermore, newly differentiated fat cells provide a monolayer culture and can be used for studies of long-term regulation of adipocyte functions [14].

### 2.2. Use of Differentiated Stroma-Vascular Cells

**Figure 1:**
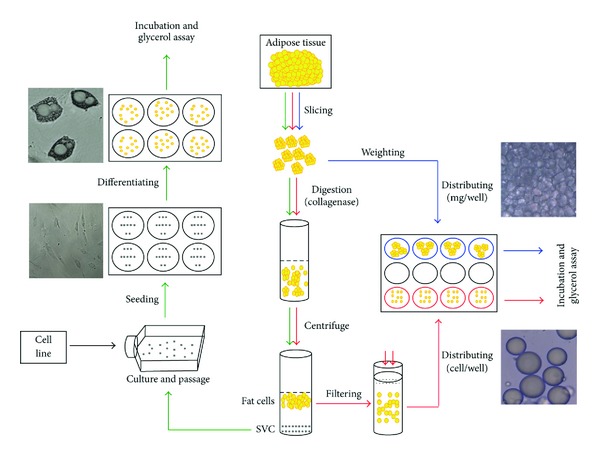
Different methods for studying lipolysis. Successive steps for organ culture of adipose tissue, primary culture of adipocytes, and differentiation of stroma-vascular cells (SVC) are shown with blue, red, and green pathways, respectively.

Adipocytes are responsible for only 25% of the total cell population of fat tissue. Therefore, nonfat cells comprise a significant part of adipose mass. This part is known as stroma-vascular fraction and includes fibroblasts, macrophages, endothelial cells, blood cells, pericytes, preadipocytes, and mesenchymal stem cells [15]. Culture of stroma-vascular cells in adipocyte differentiation medium gives rise to accumulation of lipid droplets in preadipocytes and mesenchymal stem cells [16]. Then, the differentiated adipocytes can be used for lipolysis studies (Figure 1, green pathway). Use of differentiated stroma-vascular cells, in general, has similar advantages and limitations compared to studies on preadipocyte cell lines. Yet, because stroma-vascular fraction contains various cell types, this model may mimic *in vivo* conditions of fat tissue complexity more closely than method of preadipocyte cell line culture [13].

### 2.3. Primary Culture of Adipocyte

Primary culture of fat cells was first described by Rodbell [17]. In this procedure, samples of adipose tissue removed from human or animals are sliced to small fragments and digested with collagenase. After centrifuging, the floated adipocytes are collected and incubated in the presence of tested compounds in an appropriate buffer, usually Krebs-Ringer bicarbonate buffer (Figure 1, red pathway) [18]. 

The chief advantage of this method is the ability to study lipolysis in fully differentiated adipocytes [19]. Also, the utility of primary cells provides a rapid technique for testing acute effect of a compound on lipolysis. On the other hand, use of primary adipocytes is limited by their inherent senescence and by interindividual variations [7]. Since crude collagenase may affect cell membrane function, any one series of studies must be done with the same batch of this enzyme [20]. Yet, freshly prepared adipocytes may exhibit a wide day-to-day range of lipolysis following their isolation. This problem can be ameliorated by adding adenosine deaminase to the medium to negate antilipolytic effect of endogenous adenosine [21]. In addition, long-term culture of isolated adipocytes may be associated with loss of insulin sensitivity and loss of adipocyte-specific gene expression, for example, GLUT4 [22, 23]. Because of several reports on fat depot-related variations in the hormone receptor expression, adipokine secretory profile, adipocyte cell size, glucose uptake, and lipolysis, suitable depot should be selected for adipocyte isolation according to the goal of study [24–28]. For example, it has been reported that subcutaneous adipocytes, relative to intra-abdominal adipocytes, are more responsive to antilipolytic action of insulin and less sensitive to lipolytic action of catecholamines [29–31].

### 2.4. Organ Culture of Adipose Tissue

Organ culture system of adipose tissue was first described by Slavin and Elias [32]. In this method, samples of adipose tissue are sliced into small pieces and distributed into a culture plate containing nutrients and electrolytes (e.g., M199 medium) and maintained in incubator (Figure 1, blue pathway) [33–35]. Although Gesta and coworkers showed that culture of adipose tissue leads to alteration of adipocyte gene expression, several studies demonstrated that adipose tissue can be cultured *in vitro *with preservation of hormonal responsiveness and maintenance of gene expression up to 2 weeks [36–39]. Lipolysis study can be done on organ cultured tissue itself or on adipocytes isolated from the cultured tissue [40–43].

Organ culture system of adipose tissue preserves extracellular matrix and paracrine interactions between different cell types that can influence adipocyte metabolism. Thus, data obtained from this method have a good correlation with those of *in vivo* situation and is useful for chronic lipolysis studies. Even, for acute studies, tissues can be culture 24–48 h prior to the treatments, allowing the factors to equilibrate with culture medium and therefore to minimize interindividual variability caused by subject factors such as current health status, hormonal status, and medications [33, 34, 40]. The elimination of interindividual variability causes good reproducibility in results. Further, a number of researchers have succeeded in eliciting effects of compounds using organ culture system in cases in which isolated fat cells were unresponsive [15, 19, 33, 34]. Nevertheless, with organ culture, it cannot be determined whether lipolytic or antilipolytic effects of compounds added to culture medium induced by direct actions of them on adipocyte itself or mediated by other cell types [19].

In organ culture method, like primary culture of adipocyte, suitable fat depot should be selected for studying lipolysis when considering depot-related variations in adipocyte responsiveness [24–28].

### 2.5. Microdialysis of Adipose Tissue

Microdialysis technique was introduced over 40 years ago to measure brain neurotransmitters in animal studies. This method was then adapted for adipose tissue to continuous sampling of metabolites (glucose, glycerol, lactate, adenosine, etc.) from extracellular space from animals or human. For adipose tissue microdialysis, especial probes are inserted percutaneously after light intradermal anesthesia into the subcutaneous fat. The probes are connected to a microinjection pump and perfused with Ringer solution supplemented with ethanol (for monitoring local blood flow). For lipolysis evaluation, glycerol concentration in the dialysate is determined and plotted against the perfusion rates [44, 45]. Although quantitative evaluation of glycerol release is difficult with this technique, it is a powerful method for pharmacological studies of lipolysis when used in a semiquantitative way [46]. Microdialysis permits introduction of exogenous chemicals in fat tissue to investigate the resulting local lipolysis changes without general effects on the body. In addition, this method allows continuous study of local response inside the tissue after systemic administration of chemicals [45]. It is also a promising tool in pharmacokinetic studies of drugs affecting adipose tissue metabolism [47]. One of the major disadvantages of microdialysis techniques is that its application on human is restricted to subcutaneous fat depot and it is difficult to evaluate metabolism of intra-abdominal adipose tissues [46]. Further, microdialysis is time consuming and uncomfortable for the patient and maybe associated with risk of infection. Also, various factors (perfusion rate, composition of the perfusate, size of dialysis membrane's pores, temperature inside and outside the probe, etc.) can impact on microdialysis results and the examiner should be expert in controlling these variables. 

## 3. Conclusion

Given the recent increase in attention to study the adipose tissue metabolism, this review was undertaken to compare advantages and limitations of current research methods for studying lipolysis. Several experimental techniques of adipocytes and adipose culture have been described in the litterateur. The techniques are classified into five main categories: culture of preadipocyte cell lines, use of differentiated stroma-vascular cells, primary culture of freshly isolated adipocyte, organ culture of adipose tissue, and microdialysis technique. Use of preadipocyte cell lines, differentiated stroma-vascular cells, or freshly isolated adipocytes provides abundant supply of fat cells which is suitable for screening substances for their lipolytic or antilipolytic activities and for study molecular mechanisms of lipolysis. Therefore, when the possible effect of a substance is tested for the first time, these methods are preferred. Nevertheless, cell lines or isolated cells may not exactly represent the responsiveness and the full spectrum of metabolic characteristics of adipose tissue [8]. On the other hand, organ culture and microdialysis preserve extracellular matrix and paracrine interactions and data obtained from these techniques have good correlation with *in vivo* studies. 

Taken together, this present review provides an initial comparison of different *in vitro* and *in situ* methods for studying lipolysis in the hope that chose of appropriate method for a given study aim will be considered. 

## Figures and Tables

**Table 1:** Advantages and limitations of some experimental methods for studying lipolysis.

Method	Advantages	Limitations
Culture of preadipocyte cell lines and differentiated stroma-vascular cells	Provides abundant supply of homogeneous cells Suitable for screening agents for their possible lipolytic or antilipolytic activities Appropriate for long-term *in vitro *studies Suitable for studying molecular mechanisms and transcription factors involved in lipolysis process	It takes several days until the cells become ready for study The cells may not fully differentiate to adipocytes Differentiation capacity decreases with increasing number of passages Excludes nervous system and endocrine system effects, therefore results may not fully reflect *in vivo* condition Glycerol reagent should be highly sensitive

Primary culture of adipocyte	Most of isolated adipocytes are fully differentiated A rapid method for testing acute effect of compounds Suitable for screening agents for their possible lipolytic or antilipolytic activities Comparative use of adipocytes from different depots	Need to high *n* number to resolve interindividual variations Need for enzymatic digestion Loss of hormone sensitivity and gene expression in long-term culture High variability in results obtained from freshly isolated cells Excludes nervous system and endocrine system effects Glycerol reagent should be highly sensitive

Organ culture of adipose tissue	Preserves extracellular matrix and paracrine interactions Good correlation with *in vivo* studies Useful for chronic lipolysis studies Suitable for assessing the long-term regulation of gen involved in the lipolysis pathways Comparative use of adipocytes from different depots	Presence of multiple cell types in tissue complicates interpretation of molecular mechanisms of tested drug Excludes nervous system and endocrine system effects

Microdialysis of adipose tissue	Preserves extracellular matrix and paracrine interactions Preserves nervous system and endocrine system effects Enables continuous monitoring of lipolysis Allows study of adipose tissue response during systemic drug administration	Its application on human is restricted to subcutaneous fat depot It is time consuming and uncomfortable for the patients
